# Evaluation of exogenous residual contamination in Traditional Chinese Medicine formula granules and the three-dimensional risk assessment using HI-P-CV method

**DOI:** 10.3389/fphar.2026.1730430

**Published:** 2026-05-28

**Authors:** Lin Lin, Guangzhen Liu, Lejun Tan, Fengrui Yu, Guixue Mei, Yongqiang Lin

**Affiliations:** 1 Shandong Institute for Food and Drug Control, Jinan, China; 2 Shandong University of Traditional Chinese Medicine, Jinan, China; 3 National Institutes for Food and Drug Control, Beijing, China

**Keywords:** GC-MS, hazard index method, heavy metals, HI-P-CV three-dimensional evaluation, HPLC-MS, ICP-MS, mycotoxins, pesticides

## Abstract

The residues of heavy metals, pesticides and mycotoxins in 42 types of traditional Chinese medicine formula granules (TCMFGs) were determined by inductively coupled plasma mass spectrometry (ICP-MS), high-performance liquid chromatography tandem mass spectrometry (HPLC-MS) and gas chromatography tandem mass spectrometry (GC-MS). The results showed that the detection rate of seven types of heavy metals was 100%. Among the 143 types of pesticides, 78 were detected, with an average detection rate of 24.72%. Of the 57 types of mycotoxins, 14 were detected, with an average detection rate of 38.30%. Using the hazard index method, a three-dimensional risk assessment framework based on hazard index (HI), prevalence (P), and variability (CV) (HI-P-CV framework) is established. The HI values were 0.73 for heavy metals, 0.03 for pesticides and 0.54 for mycotoxins, individually. The CV values were 59.15%, 59.54% and 42.57%, respectively. The model revealed significant variation in contamination levels among three categories of residues in TCMFGs, indicating hidden risks. Cumulative dietary risk assessment identified 18 varieties with potential health risks, including 10 high-risk varieties. The average cumulative dietary risk was 1.3, indicating a relatively high overall risk of exogenous residue contamination in TCMFGs. Profile analysis indicated that varieties with aerial medicinal parts tend to accumulate heavy metals and should be prioritized for heavy metal contamination monitoring. Varieties with underground medicinal parts, which are rich in polysaccharides, are more susceptible to fungi contamination. The detection of banned pesticides and widespread presence of plant growth regulators highlight the need for stricter regulation of pesticide use.

## 1 Introduction

With the acceleration of modern life, chronic diseases and sub-health issues have become increasingly common. People are turning back to natural therapies, and the efficacy of traditional Chinese medicines (TCMs) has gained widespread recognition in both domestic and international markets. The World Health Organization (WHO) has pointed out that 75% of the global population uses herbal medicines to meet basic healthcare needs (Pan et al., 2014). Meanwhile, as the global acceptance of TCMs continues to grow, their applications have also expanded to other fields, such as health products and dietary supplements (Piemontese, 2017), etc. Renowned pharmaceutical companies equipped with modern technology have begun to re-explore herbs as a potential source of new medicine candidates, supporting the development and discovery of natural product-based medicines (Li and Zhang, 2008; Corson and Crews, 2007; Schmidt et al., 2007).

As demand for TCMs increases and wild herbal resources become increasingly scarce, artificial cultivation has become the main source of medicinal herbs. However, during cultivation, processing, and storage, TCMs are prone to contamination by exogenous pollutants, mainly including heavy metals, pesticides, and mycotoxins, etc. (Steinhoff, 2019; Bortey-Sam et al., 2015; Gündüz, 2015; Kashyap et al., 2018; Zhang et al., 2016; Seidl, 2002). These exogenous residues can seriously affect the quality and safety of TCMs, which is a major concern in the international market.

The traditional Chinese medicine formula granules (TCMFGs) are pure herbal products made from single-ingredient herbal decoctions that meet processing standards. They are produced using modern pharmaceutical techniques such as extraction, concentration, separation, drying, granulation, and packaging. As an innovative modern dosage form, TCMFGs offer advantages such as stable quality and convenient administration, and their use is expanding both domestically and internationally, with growing market share.

Due to their more complex production process, TCMFGs are more prone to contamination by exogenous residues compared to traditional herbal decoctions. For example, organochlorine pesticides, due to their stable chemical properties, may still be detected in TCMs even years after being banned and can become concentrated during the extraction process. Additionally, since TCMFGs require water decoction to extract active ingredients, some lipophilic pesticides and mycotoxins, which are low solubility in water, may remain and even become concentrated by over tenfold during the concentration process. Furthermore, if production water or equipment is contaminated (e.g., heavy metals leaching from pipes), new exogenous harmful substances may be introduced. Therefore, comprehensive screening and risk assessment of exogenous harmful residues in TCMFGs are necessary to ensure their safety.

As a new type of herbal decoction, TCMFGs are subject to the same exogenous residue control requirements as TCMs. Currently, research on controlling exogenous residues in TCMs mainly focuses on optimizing testing methods and establishing risk assessment systems. The 2025 edition of the Chinese Pharmacopoeia (ChP, 2025) provides, in General Chapters 2,321, 2,341, 2,342, and 2,351, methods for the determination of heavy metals, pesticide residues, plant growth regulator residues, and mycotoxins in TCMs. Guideline 9,302 in ChP 2025, which is Guidelines for the Establishment of Limits for Harmful Residues in Traditional Chinese Medicine, offers theoretical foundations, calculation methods, and influencing factors for setting maximum limits of harmful residues in TCMs. In contrast, Europe, the United States, Japan, and other countries and regions have stricter regulations on herbal medicines, having established relatively comprehensive quality and safety monitoring systems. They widely adopt stricter limits and testing methods developed by the Codex Alimentarius Commission (CAC), the U.S. Food and Drug Administration (FDA), and the European Food Safety Authority (EFSA).

At present, most exogenous residue control indicators for TCMs lack maximum residue limits (MRLs), making it difficult to determine whether a product exceeds safety thresholds. Moreover, MRLs are product-based limits, not necessarily safety limits (Wanwimolruk et al., 2015). A product exceeding the recommended MRL is not necessarily unsafe. In some cases, consumer intake levels and frequency of use are important factors influencing risk assessment conclusions. Risk assessment is a scientific process of evaluating the potential adverse health effects of biological, chemical, and physical hazards in the environment, food, and medicine. It is also a key step in determining the potential health risks posed by exogenous harmful residues in TCMs. The ultimate goal of risk assessment is to identify high-risk products and provide scientific regulatory recommendations to risk managers.

In recent years, researchers have paid increasing attention to systematic evaluation and risk analysis of TCMs (Luo et al., 2021; Xiao J. et al., 2018; Xiao J. J. et al., 2018; Wang et al., 2022; Zuo et al., 2025), using hazard quotient and index methods to assess potential health risks (Wu et al., 2020). The objective of this study is to understand the contamination status of exogenous residues in TCMFGs and to evaluate their health exposure risks. The residues of heavy metals, pesticides and mycotoxins in 42 types of TCMFGs were determined by inductively coupled plasma mass spectrometry (ICP-MS), high-performance liquid chromatography tandem mass spectrometry (HPLC-MS) and gas chromatography tandem mass spectrometry (GC-MS). Using the hazard index method, a three-dimensional risk assessment framework based on hazard index (HI), prevalence (P), and coefficient of variation (CV) (HI-P-CV framework) is established. This study explores and proposes a risk assessment model suitable for TCMFGs, providing monitoring recommendations for the industry.

## 2 Materials and methods

### 2.1 Materials and reagents

Heavy metal and harmful element standards were provided by Guobiao (Beijing) Testing & Certification Co., Ltd. Pesticide, plant growth regulator and mycotoxin standards were provided by the Ministry of Agriculture (Beijing, China), the National Institute for Food and Drug Control, and Dr. Ehrenstorfer GmbH, and all had >96% purity. QuEChERS extraction packs (containing 6 g anhydrous MgSO_4_, 1.5 g anhydrous CH_3_COONa) and silica gel dispersive purge tubes (containing 100 mg C18, 100 mg PSA, 90 mg GC-e, and 300 mg anhydrous MgSO_4_) used for dispersive solid-phase extraction analysis were from Shimadzu (Japan). Analytical glacial acetic acid, and solvents were provided by Sinopharm Chemical Reagent Co., Ltd (Shanghai, China). Superior grade pure nitric acid, glacial acetic acid, formic acid, ammonium formate and HPLC grade acetonitrile were from Fisher Scientific (United States).

Individual 1,000 μg/mL heavy metal and harmful element stock solutions were prepared in 1%–5% HNO_3_, and stored at −20 °C until analysis. Mixtures of working standard solutions at a series of concentrations were made by diluting aliquots of the stock mixture in 2% HNO_3_. Individual 100 μg/mL pesticide, plant growth regulator and mycotoxin stock solutions were prepared in methanol, acetonitrile, toluene or acetone, and stored at −20 °C until analysis. Mixtures of working standard solutions at a series of concentrations were made by diluting aliquots of the stock mixture in acetone.

### 2.2 Sample collection

A total of 420 samples were collected, covering 42 varieties, with 10 batches for each variety. The 42 varieties include Bletillae Rhizoma (BR), Paeoniae Radix Alba (PRA), Atractylodis Macrocephalae Rhizoma (AMR), Angelicae Dahuricae Radix (ADR), Bupleuri Radix (BUR), Chuanxiong Rhizoma (CHR), Salviae Miltiorrhi-Zae Radix Et Rhizoma (SMRR), Angelicae Sinensis Radix (ASR), Codonopsis Radix (CR), Glycyrrhizae Radix Et Rhizoma (GRER), Ginseng Radix Et Rhizoma Rubra (GRERR), Polygonati Rhizoma (PRH), Astragali Radix (AR), Ophiopogonis Radix (OR), Achyranthis Bidentatae Radix (ABR), Ginseng Radix Et Rhizoma (GRR), Dioscoreae Rhizoma (DR), Trichosanthis Radix (TR), Linderae Radix (LR), Panacis Quinquefolii Radix (PQR), Scrophulariae Radix (SR), Polygalae Radix (PR), Arecae Semen (AS), Jujubae Fructus (JF), Lycii Fructus (LF), Nelumbinis Foliu (NF), Corni Fructus (COF), Crataegi Fructus (CRF), Persicae Semen (PES), Mume Fructus (MF), Prunellae Spica (PRS), Lonicerae Japonicae Flos (LJF), Coicis Semen (CS), Gardeniae Fructus (GF), Rosae Rugosae Flos (RRF), Buddlejae Flos (BF), Trachelospermi Caulis Et Folium (TCEF), Artemisiae Annuae Herba (AAH), Cirsii Herba (CH), Citri Reticulatae Pericarpium (CRP), Nelumbinis Semen (NS), Moutan Cortex (MC). All samples were purchased from various medical institutions. Samples were kept at below 20 °C until analysis.

### 2.3 Sample preparation

#### 2.3.1 Pretreatment for heavy metal and harmful element

Accurately weigh approximately 0.5 g of the sample and place it into a polytetrafluoroethylene digestion vessel. Add 5 mL of nitric acid and soak overnight. Then, carry out microwave digestion. After cooling the digest to below 60 °C, transfer it to a 50 mL volumetric flask. Rinse the digestion vessel with a small amount of water, combine the rinsing liquid into the flask, add 200 μL of gold single-element standard solution (1 μg/mL), dilute to the mark with water, and mix well to obtain the test solution.

#### 2.3.2 Pretreatment for pesticide, plant growth regulator and mycotoxin

Accurately weigh 3 g of sample powder into a 50 mL centrifuge tube, add 15 mL of deionized water (containing 1% acetic acid), and vortex to mix. Incubate at room temperature for 30 min, then add 15 mL of acetonitrile and vortex for 5 min. Immediately cool in an ice-water bath for 30 min. Add 6 g of MgSO_4_ and 1.5 g of anhydrous CH_3_COONa, then vortex the tube vigorously for 5 min and cool in an ice-water bath for another 10 min. Centrifuge the tube at 8,000 rpm for 5 min at 8 °C to separate the two phases. For further cleanup, transfer 8 mL of the supernatant into a 15 mL QuEChERS silica gel dispersive purge tubes. Vortex the mixture vigorously for 5 min and centrifuge at 8,000 rpm for 5 min. Finally, filter the supernatant through a 0.22 μm nylon organic filter for analysis.

### 2.4 Preparation of calibration curves

Method 1 (for heavy metals and harmful elements): Two calibration ranges were designed. Low-level curve: intended for analytes expected near the LOQ. The range spans from the estimated limit of detection to a moderate concentration and comprises 0.0005, 0.001, 0.005, 0.01, 0.05 and 0.1 mg/kg. This curve ensures optimal linearity and accuracy at ultra-trace levels. High-level curve: intended for elements present at higher concentrations. The range spans from the estimated limit of detection to a moderate concentration and comprises 0.05, 0.1, 0.5, 1.0, 5.0 and 10.0 mg/kg. This curve is also used to verify results when the concentration of a low-level analyte exceeds the upper limit of the low-level curve. Both curves were prepared by serial dilution of a certified multi-element high-concentration stock solution in 10% HNO_3_ containing internal standards. A linear regression model with a 1/x weighting factor was applied to improve the fit across the dynamic range.

Method 2 (for pesticides, plant-growth regulators and mycotoxins): Blank samples free of the target analytes were processed in the same way as the test samples to obtain a blank matrix solution. Multi-residue pesticide or mycotoxin stock mixtures were then step-wise diluted with this blank matrix to prepare calibration solutions at 0.005, 0.010, 0.020, 0.050, 0.100 and 0.200 mg/kg. A linear regression model with a 1/x weighting factor was again used to enhance linearity.

### 2.5 Sample analysis

#### 2.5.1 ICP-MS

A PE ICP instrument interfaced with a NexION 1,000 Triple Quad mass spectrometry system (PerkinElmer Co.,United States) was used for sample analysis. Collision gas reaction mode, inductively coupled plasma radio frequency power 1600 W, analog voltage −1700 V, pulse voltage 1200 V, plasma gas flow rate 15 L/min, atomizer flow rate 1.01 L/min, collision gas flow rate 3.5 L/min. During the determination, for ^51^V, ^58^Ni, ^59^Co, ^45^Sc is used as the internal standard, for ^75^As, ^72^Ge is used as the internal standard, for ^114^Cd, ^115^In is used as the internal standard, for ^202^Hg and ^208^Pb, ^209^Bi is used as the internal standard.

#### 2.5.2 UPLC-MS/MS

A Shimadzu UHPLC instrument interfaced with a 8,050 Triple Quad mass spectrometry system (Shimadzu Co., Japan) was used for sample analysis. Separation was carried out on a Poroshell 120 EC-C18 column (3.0 × 150 mm, 2.7 μm, Agilent). The mobile phase includes solution A (2 mmol/L ammonium formate and 0.01% formic acid in methanol) and B (2 mmol/L ammonium formate and 0.01% formic acid in water). The following gradient was applied at a flow rate of 0.3 mL/min: 0–1.5 min, 3%–15% A; 1.5–2.5 min, 15%–50% A; 2.5–18.0 min, 50%–70% A; 18.0–23.0 min, 70%–98% A; 23.0–27.0 min, 98% A. The injection volume was 1 μL. Mass spectrometry was performed using an electrospray ionization (ESI) source operated in positive/negative switching mode under multiple reaction monitoring (MRM). The optimized ion source parameters were as follows: nebulizing gas flow, 3.0 L/min; drying gas flow, 10.0 L/min; heating gas flow, 10.0 L/min; interface temperature, 300 °C; desolvation line (DL) temperature, 150 °C; heat block temperature, 400 °C; interface voltage, 4.0 kV for ESI^+^ and −3.5 kV for ESI^−^.

#### 2.5.3 GC-MS/MS

A Shimadzu GC equipped with a Triple Quad mass spectrometer system TQ8050NX (EI source) was used to perform analysis with a DB-17M capillary column (30 m × 0.25 mm × 0.25, Agilent). The oven temperature was programmed at 50 °C for 1 min, after which it was gradually increased to 125 °C at a rate of 25 °C/min and to 300 °C at 10 °C/min, held for 10 min. The inlet temperature was 250 °C. The injection volume was 1 µL in the splitless mode. The carrier gas was helium. The injection port is in constant pressure mode, and the pressure before the column is 100 kPa. The mass spectrometer was operated in the MRM mode with nitrogen as the collision gas at a flow rate of 1.5 mL/min. The temperatures of the ion source and transfer lines were 200 °C and 250 °C, respectively. The solvent delay was set at 5.0 min.

### 2.6 Health risk assessment

#### 2.6.1 Intake risk assessments

According to the risk characterization method recommended by WHO, the HI method was used to characterize the risk profile of TCMFGs. The Hazard Quotient (HQ) was employed to assess dietary exposure risk. The assessment model was constructed based on the method recommended by the Food and Agriculture Organization (FAO) and WHO for evaluating human chemical intake levels (FAO, 2012; WHO (World Health Organization), 2016). The oral exposure levels of various exogenous residues were calculated and compared with the safety reference dose (RfD). The RfD was based on toxicological data such as ADI, TDI, BMDL, and NOAEL, sourced from WHO, EU regulations, the European Food Safety Authority (EFSA), and the U.S. Environmental Protection Agency (EPA). HQ are calculated according to formula 1:

$$HQ=\frac{R\times CR}{bw\times RfD}$$ (1)

Where R represents the average residue level in the sample (mg/kg); CR is the average consumption (kg/day); bw is the average weight of Chinese adults (63 kg) (the 2025 edition of the Chinese Pharmacopoeia (General Chapter 9,302); Wang et al., 2022). When HQ < 1, the risk is considered acceptable; when HQ > 1, the risk is considered unacceptable, with higher values indicating greater risk. During exposure assessment, the detection rate of residues in the concentration dataset is of critical importance. The processing and calculation of these values may affect the assessment results (Ooijen et al., 2009). In this study, according to WHO recommendations, residue concentrations below the Limit of Quantification (LOQ) were treated as 0.5 × LOQ, which is considered acceptable (GEMS/Food Global EnvironmentMonitoring System, 2016).

#### 2.6.2 Cumulative risk assessment

The HI is a parameter used for cumulative risk assessment (Li et al., 2018). According to Formula 2, HI is expressed as the sum of the HQ values of each residue in the sample. The HI method is clear, easy to understand, and directly related to the reference dose. As a rapid and simple approach, HI has been widely used in cumulative risk assessments of various sample types, such as air (Xiao J. et al., 2018), soil (Chang et al., 2014), food (Lyulyukin et al., 2018) and TCM (Wang et al., 2022). In this study, the HI method was used to assess the cumulative risk of exogenous residues in TCMFGs.

$$HI=\sum_{i=1}^{n}HQ$$ (2)

Where n is the total number of exogenous residues. When HI > 1, the TCMFGs involved are considered to pose a risk to consumers; when HI < 1, the TCMFGs are considered acceptable.

#### 2.6.3 HI-P-CV three-dimensional risk assessment

The HI-P-CV three-dimensional risk assessment framework consists of three dimensions: Hazard Index (HI), Prevalence (P), and Coefficient of Variation (CV). It reflects the risk index of residues, the stability of their occurrence, and the batch-to-batch variability risk. Due to the large number of residue types and detection indicators, residues are classified into three major categories: heavy metals, pesticide residues, and mycotoxins for analysis. The HI, P, and CV values for each category were calculated using Formulas 3-5:

$${\bar{HI}}_{class}=\frac{1}{N_{c}}\sum_{i=1}^{N_{c}}\left(\frac{1}{N_{h}}\sum_{j=1}^{N_{h}}HQ_{ij}\right)$$ (3)

Where ${\bar{HI}}_{class}$ is the Hazard Index of categorie of heavy metals, pesticide residues, or mycotoxins, *N*_*c*_ is the number of contaminants in the class, *N*_*h*_ is the number of TCMFG varieties, and *HQ*_*ij*_ is the HQ of contaminant *i* in TCMFG *j*.

$${\bar{P}}_{\text{class}}=\frac{1}{N_{c}}\sum_{i=1}^{N_{c}}\left(\frac{1}{N_{h}}\sum_{j=1}^{N_{h}}P_{ij}\right)$$ (4)

Where ${\bar{P}}_{\text{class}}$ is the prevalence of categorie of heavy metals, pesticide residues, or mycotoxins, *N*_*c*_ is the number of contaminants in the class, *N*_*h*_ is the number of TCMFG varieties, and *P*_*ij*_ is the detection rate of contaminant *i* in TCMFG *j*.

$${\bar{CV}}_{\text{class}}=\frac{\sum_{i=1}^{N_{c}}\sum_{j=1}^{N_{h}}\left(CV_{ij}\times {\bar{C}}_{ij}\right)}{\sum_{i=1}^{N_{c}}\sum_{j=1}^{N_{h}}{\bar{C}}_{ij}}$$ (5)

Where ${\bar{CV}}_{\text{class}}$ is the variability of categorie of heavy metals, pesticide residues, or mycotoxins, *N*_*c*_ is the number of contaminants in the class, *N*_*h*_ is the number of TCMFG varieties, *CV*_*ij*_ and $\bar{C}{}_{\text{ij}}$ is the coefficient of variation and mean concentration of contaminant *i* in TCMFG *j*, respectively. This weighting approach accounts for the greater impact of high-concentration contaminants on overall risk.

## 3 Results

### 3.1 Method validation

The in-house validation data was achieved by evaluating the following parameters: Limit of Quantification (LOQ), precision, linearity, and accuracy. The LOQ for heavy metals was calculated from the standard deviation of the linear response and the slope. For pesticides and mycotoxins, the LOQ was determined by the signal-to-noise approach, i.e., the LOQ for each analyte was defined as the lowest concentration of the target compound that produced a detectable signal. Accuracy and precision were evaluated through recovery experiments at three spiking levels (LOQ, 5×LOQ, and 10×LOQ). Matrix-matched calibration curves were used to establish the linear range for pesticide and mycotoxin analyses. All residues showed good linearity within the relevant concentration range, with correlation coefficients (*r* ^*2*^) greater than 0.995. The recovery rates of all detected residues ranged from 65% to 120%, with relative standard deviations (RSD) ≤ 20%.

### 3.2 Residual contamination status

#### 3.2.1 Selection of varieties and detection indexes

A total of 42 TCMFG varieties with the highest market circulation volume were selected. High circulation implies extensive clinical use, so the selection is highly relevant. Samples were collected from nine manufacturers in Beijing, Shandong, Jiangsu, Sichuan and Guangdong, giving good geographical representation. The 42 varieties cover roots and rhizomes, fruits, flowers, seeds, fruit spikes, fruit peels, root barks, leaves, leafy vines and aerial parts, providing broad representation of medicinal organs. These varieties are therefore well suited to reflect the contamination status of exogenous residues in TCMFGs.

For heavy metals the ICH-Q3D Class-1 elements (As, Cd, Hg, Pb) and Class-2A elements (Co., Ni, V) were chosen. Pesticides were selected on the basis of the Ministry of Agriculture’s guidelines on highly toxic/persistent pesticides, market surveys and field investigations; 104 traditional pesticides and 39 plant-growth regulators were included, covering most banned pesticides in China plus those commonly used in herbal cultivation. Mycotoxins were chosen according to the ChP2025 guidelines 9,305 (ChP, 2025), 57 mycotoxins were targeted, including most fungi likely to contaminate medicinal plants.

#### 3.2.2 Heavy metal residues identified

**FIGURE 1:**
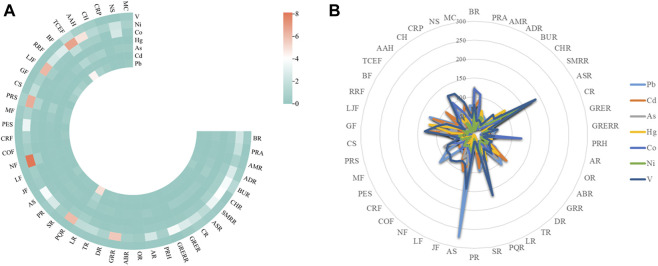
The average residues of heavy metal in each TCMFG **(A)** represents the mean value, and **(B)** represents the CV value.

Among the seven types of heavy metals tested, the detection rate in 420 samples was 100%. Such a high detection rate is closely related to the LOQs: apart from nickel, whose LOQ is about 0.100 mg/kg, the LOQs for the other six heavy metals are all below 0.001 mg/kg. Except for a few individual varieties and indices, most measured values are low. The distribution of average residue levels across different varieties is shown in Figure 1. These results indicate that the overall heavy metal contamination level of TCMFGs is not high, but certain varieties and elements may exhibit accumulation characteristics. The concentration ranges, average values, coefficients of variation, and detection rates of each indicator are provided in the Supplementary Material S1.

#### 3.2.3 Pesticide residues identified

**FIGURE 2:**
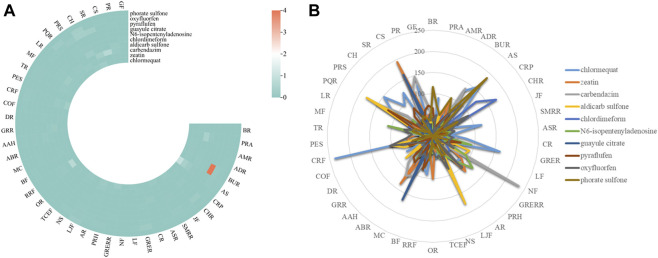
The average residues of pesticide in each TCMFG **(A)** represents the mean value, and **(B)** represents the CV value.

Among the 143 compounds tested, 78 were detected in 420 samples, including 46 traditional pesticides and 32 plant-growth regulators. Twenty-seven compounds were found in more than 30% of the samples; the ten most frequently detected were oxyfluorfen (93.6%), guayule citrate (68.8%), zeatin and pyraflufen (63.3%), chlordimeform (61.9%), N6-isopentenyladenosine and phorate sulfone (56.2%), carbendazim and aldicarb sulfone (55.7%), and chlormequat (54.5%). Among these, five were conventional pesticides and five were plant-growth regulators. The average residue distribution of variety is shown in Figure 2. The most frequently detected compound, oxyfluorfen, is a widely used herbicide characterised by a broad weed spectrum, long residual activity, low application rate, high efficacy and low toxicity. The concentration range, average values, coefficient of variation and detection rate of each detection index are provided in the Supplementary Material S1.

Compared with traditional Chinese herbal decoction pieces, the detection rate of pesticide residues in TCMFGs is significantly higher. In traditional decoction pieces, the pesticides most frequently detected are carbendazim (35.7%), chlorpyrifos (34.1%), paclobutrazol (26.7%), difenoconazole (20.5%), tebuconazole (18.5%), acetamiprid (17.7%), carbofuran (17.0%), cypermethrin (16.0%), imidacloprid (15.0%), and pentachloronitrobenzene (14.6%) (Wang et al., 2022). The corresponding detection rates in formula granules are carbendazim (55.7%), paclobutrazol (20.5%), difenoconazole (32.1%), tebuconazole (35.2%), acetamiprid (24.3%), carbofuran (10.5%), and imidacloprid (41.2%). This discrepancy is likely related to the manufacturing process of formula granules: extraction, concentration, and drying remove large amounts of plant fiber and other materials. While a few pesticides fail to transfer into the decoction because of their limited solubility, most residues are concentrated into the much smaller granule mass, resulting in a higher pesticide concentration per unit weight in the final product and making them easier to detect. Among the 46 conventional pesticides detected, 19 are officially banned, indicating either continued illicit use or persistence of historically applied residues that remain bio-available in soil. The detection rates of five pesticides exceeded 30%, with chlordimeform (61.9%), phorate sulfone (56.2%), fonofos (39.8%), difluoroatorvastatin (36.7%) and posfolan-methyl (33.1%), which should be given sufficient attention.

#### 3.2.4 Mycotoxin residues identified

**FIGURE 3:**
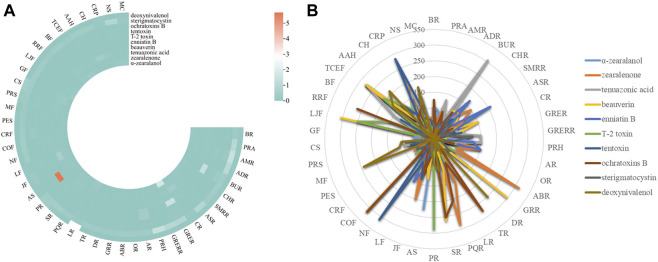
The average residues of mycotoxin in each TCMFG **(A)** represents the mean value, and **(B)** represents the CV value.

Of the 57 mycotoxins tested, 14 were detected in 420 samples. The ten most frequently detected mycotoxins were sterigmatocystin (81.7%), zearalenone and deoxynivalenol (41.9%), T-2 toxin (40.0%), enniatin B (38.6%), tenuazonic acid (38.1%), α-zearalanol (37.4%), tentoxin (36.9%), beauvericin (35.7%) and ochratoxin B (33.6%). The average residue distribution by variety is shown in Figure 3. Sterigmatocystin, the most prevalent, is carcinogenic and teratogenic and is classified as a Group 2B human carcinogen by International Agency for Research on Cancer (IARC); it is strongly associated with lung, liver and gastric cancers and therefore requires special attention. The concentration range, average values, coefficient of variation and detection rate of each detection index are provided in the Supplementary Material S1.

#### 3.2.5 Compliance analysis

**FIGURE 4:**
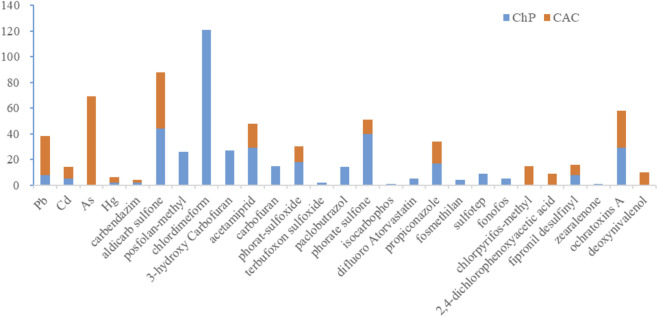
Compliance analysis of pollutants and residues.

According to ChP 2025 and CAC (Codex Alimentarius Commission, 2024; Codex Alimentarius Commission, 2025), a compliance analysis was conducted on the detected heavy metals, pesticide residues, and mycotoxins. Among them, 27 types of pollutants and residues were found to be non-compliant, as shown in Figure 4. Since CAC serves as the benchmark standard for international food trade, its formulation requires consensus among all member countries. The indicators of pollutants and residues are relatively few. While ChP 2025 only stipulates specific limit standards for some Chinese medicinal herb varieties, most pollutants do not have general limit standards. When expanding the scope for reference, a considerable portion of pollutants and residues still have no reference limit regulations in both CAC and ChP 2025. In the future, continuous attention should be paid to the update of domestic and international standards, and efforts should be made to promote the construction of a pollutant limit standard system specific to TCMs.

### 3.3 Health risk assessment

#### 3.3.1 Intake risk assessment

**FIGURE 5:**
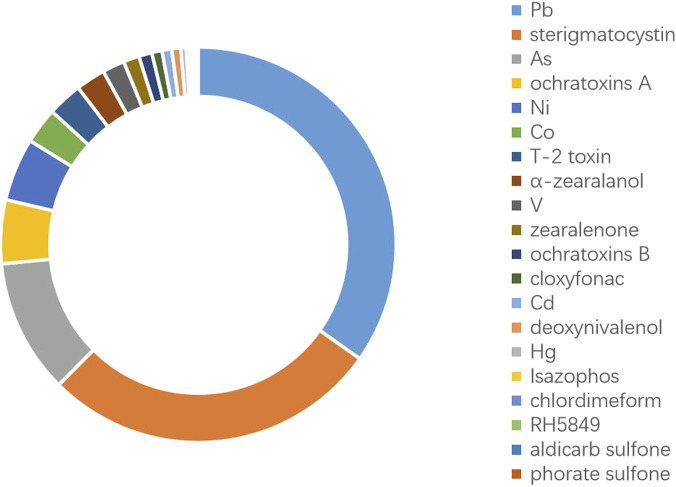
The 20 compounds with HQ > 0.002.

**FIGURE 6:**
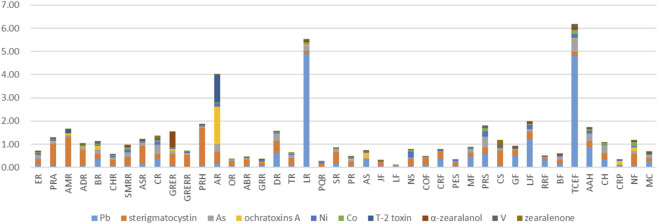
The distribution of the top 10 compounds in the HQ ranking in TCMFG.

Health-based guidance values are available for 79 of the 99 compounds detected (7 types of heavy metals, 78 types of pesticides, and 14 types of mycotoxins). The HQ values were calculated using mean concentrations (Supplementary Material S2). The majority of compounds posed low intake risk (median HQ = 0.0002). The 20 compounds with HQ > 0.002 are shown in Figure 5; the top 10 contributors accounted for >95% of the total intake risk, and their distribution across the 42 varieties is presented in Figure 6.

#### 3.3.2 Cumulative dietary risk assessment

**FIGURE 7:**
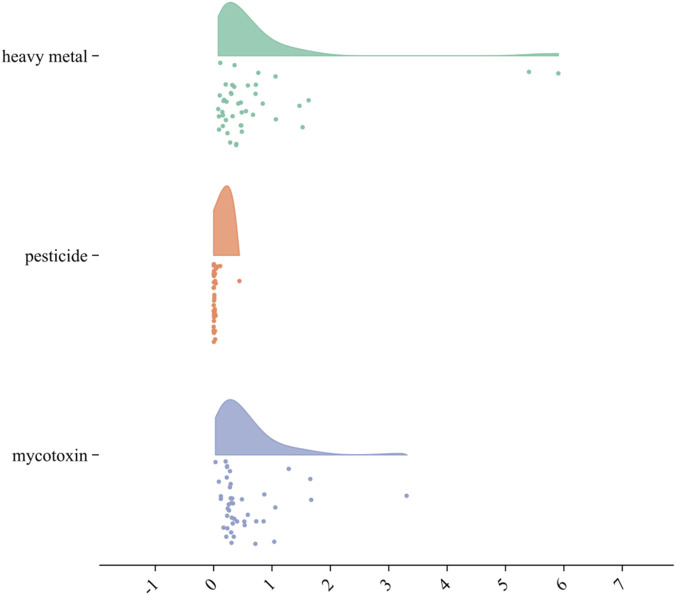
The cumulative dietary risks of heavy metals, pesticide residues and mycotoxins.

Simple additive HI were calculated separately for heavy metals, pesticides and mycotoxins in each of the 42 varieties. The data were right-skewed, with most values clustered at the low end and few at the high end. Seven varieties exceeded an HI of 1 for heavy metals, whereas none did for pesticides/plant-growth regulators; seven varieties exceeded an HI of 1 for mycotoxins (Figure 7). The average cumulative dietary risk values were 0.73 for heavy metals, 0.03 for pesticides and 0.54 for mycotoxins, indicating generally low risk from each contaminant category individually.

**FIGURE 8:**
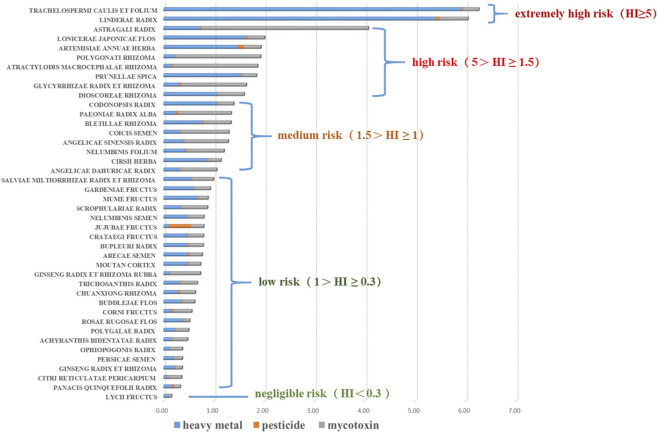
The HI of heavy metal, pesticide, mycotoxin and the sum in each TCMFG.

Without considering synergism, simple summation of HQ values for heavy metals, pesticides and mycotoxins showed 18 varieties with total HI > 1. The 42 varieties were classified into five groups (Figure 8): two extremely high-risk (TCEF, LR), eight high-risk (AR, DR, LJF, AAH, PRH, AMR, PRS, GRER), eight moderate-risk, and 24 low and negligible-risk. The average dietary risk was 1.30, indicating that exogenous residues in TCMFGs pose an overall risk.

In cumulative risk assessment, a hazard index (HI) of ≥1 theoretically indicates that the exposure level exceeds the safety reference value, suggesting potential health risks. However, in practice, a higher threshold is usually set to distinguish between theoretical risks and those that require immediate attention. This study analyzed the distribution of HI values for 42 types of traditional Chinese medicine formula granules. If the high-risk threshold is set as HI ≥ 1, 16 varieties (38.1%) are classified as high-risk, which leads to an overgeneralization of risk signals and is not conducive to focusing on key risk points. If the high-risk threshold is set as HI ≥ 1.5, 10 varieties (23.8%) are classified as high-risk, including eight high-risk and two extremely high-risk ones. This can effectively screen out the varieties that require priority attention, which is in line with the research positioning of risk screening and priority ranking. Therefore, this study chose HI ≥ 1.5 as the high-risk threshold, and using this threshold can effectively screen out the top 10 varieties as high-risk focus objects, which is in line with the research positioning of risk screening.

#### 3.3.3 HI-P-CV risk assessment

**FIGURE 9:**
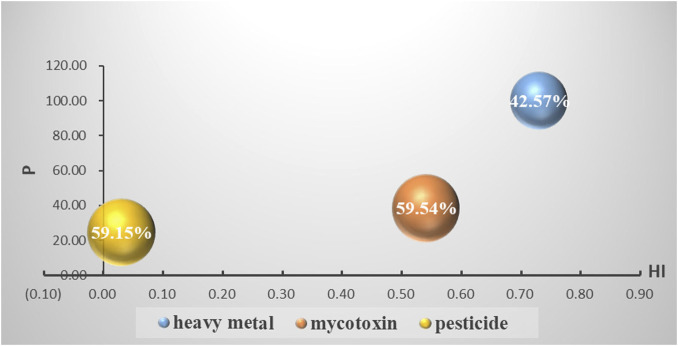
HI-P-CV risk assessment distribution bubble chart.

The ${\bar{HI}}_{\text{class}}$ , ${\bar{P}}_{\text{class}}$ , and ${\bar{CV}}_{\text{class}}$ were computed for heavy metals, pesticide residues and mycotoxins to evaluate overall contamination and health risk of exogenous residues in TCMFGs. All ${\bar{HI}}_{\text{class}}$ values were <1, implying low aggregate health risk. The average detection rates were 100% for heavy metals, 24.72% for pesticides and 38.30% for mycotoxins; the average CV values were 59.15%, 59.54% and 42.57%, respectively (Figure 9). The wide variability indicates heterogeneous contamination and highlights hidden risks, underscoring the need for strengthened quality control.

## 4 Discussion

### 4.1 Risk assessment of pesticides in different medicinal positions

The 10 medicinal parts were grouped into: underground parts (23 varieties), aerial parts (8 varieties) and fruits and seeds (11 varieties). Risk differed markedly among the different medicinal parts. Among fruits and seeds only CS showed moderate risk (HI > 1), all others were low risk. Of the 18 varieties with detectable risk, 11 belonged to underground parts (47.8% of 23) and six to aerial parts (75.0% of 8). Reproductive organs such as fruits and seeds are not primary absorptive parts and are often annual, whereas underground and aerial parts are active absorbers that readily accumulate exogenous pollutants.

### 4.2 Analysis of the top 10 risk product

**FIGURE 10:**
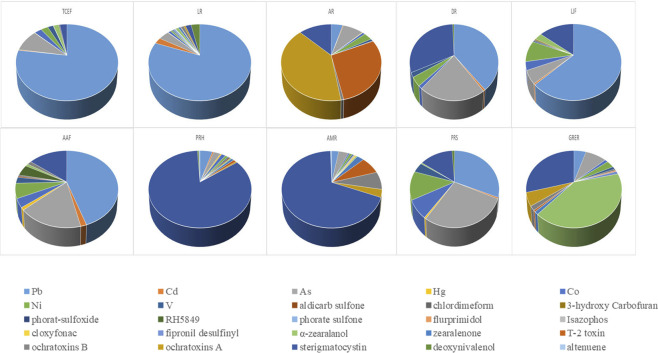
Analysis of the top 10 risk varieties.

The sources of risk for varieties with HI > 1.5 were analysed, the top 25 HQ compounds are shown in Figure 10. Lead was the dominant contaminant in six varieties (TCEF, LR, DR, LJF, AAH, PRS), including all four aerial-part varieties, suggesting that aerial parts readily accumulate lead. These four also showed notable arsenic contamination, indicating that aerial absorptive organs preferentially accumulate heavy metals. PRH and AMR were dominated by sterigmatocystin, AR by ochratoxin A and T-2 toxin, and GRER by α-zearalanol; DR also showed marked sterigmatocystin contamination. These five are underground parts rich in polysaccharides and are therefore prone to fungal attack.

### 4.3 Top 10 analysis of the distribution of the top 10 pollutants

**FIGURE 11:**
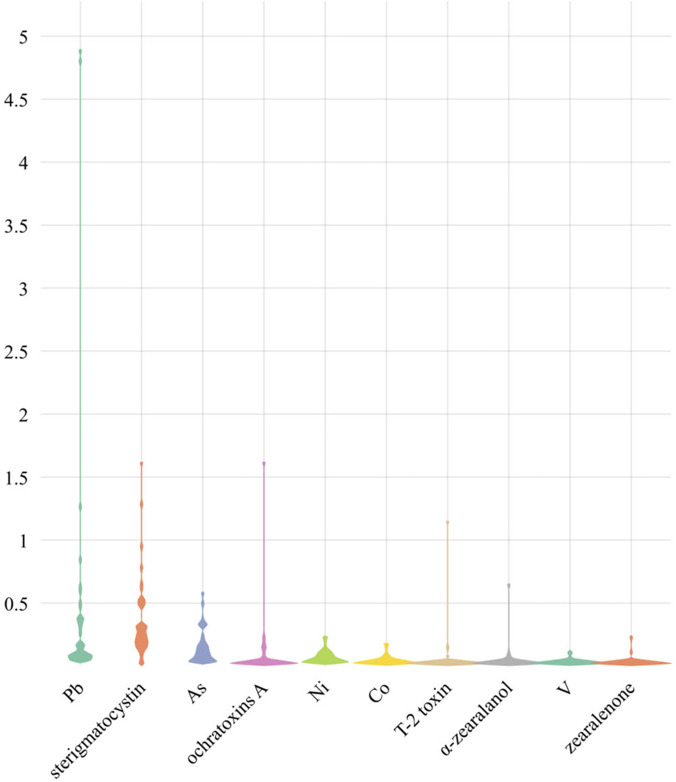
Analysis of the distribution of the top 10 pollutants.

The distribution of the top 10 pollutants in all 42 varieties is shown in Figure 11. High-density regions for all 10 pollutants lie below 0.5, indicating generally low contamination. Lead, sterigmatocystin, ochratoxin A and T-2 toxin exhibit long right tails with low dispersion, signifying a few highly contaminated samples; sources or variety-specific accumulation traits should be investigated. Nickel, cobalt and vanadium are highly clustered; equipment (stainless steel may introduce Fe, Cr, Ni), process water and packaging materials should be evaluated as sources of elemental impurities. Because Class-2A elements have relatively high oral PDE values, their risk in TCMFGs is considered low.

In the HI-P-CV risk assessment, CV was calculated by a novel stratified mean + concentration-weighted method to ensure scientific rigour. Pollutants were grouped into heavy metals, pesticides and mycotoxins for separate evaluation. Long-term exposure assessments are most accurate when reliable mean residue levels and consumption data are available. Daily intake ranges for the 42 herbs are given in the ChP 2025; ChP, 2025), the median was used as the average consumption. For residue levels, the median is unaffected by high outliers, possibly underestimating lifetime exposure to heavily contaminated batches. Because individuals will encounter all contamination levels over a lifetime, the mean provides a more realistic estimate and is preferred by EFSA and FAO/WHO (EFSA, 2009; FAO/WHO, 2011); therefore mean concentrations were used to conservatively estimate worst-case scenarios.

This study found high mean coefficients of variation (CV) for the three contaminant categories (42.57%, 59.15%, 59.54%), driven mainly by differences among batches. Inter-batch repeatability is a critical indicator of both the reliability of any analytical method and the extent to which the resulting data reflect the product’s intrinsic properties. Here, it directly affects the accuracy of the HI-P-CV model and the credibility of the final risk-assessment conclusions. Poor inter-batch repeatability is not a methodological flaw of this study, but a real and important risk attribute inherent to TCMFGs. Although the HI-P-CV model and cumulative risk assessment used here are based on overall means and already reveal a generally elevated risk, future regulatory and quality-control efforts must prioritize reducing inter-batch variability to ensure product consistency.

The HI-P-CV risk assessment of heavy metals, pesticides and mycotoxins represents the highest-tier and most realistic exposure scenario, but is also the most challenging because the three groups differ in toxic mechanisms, data availability and modelling approaches; no internationally harmonised model exists. In this study only simple HQ summation without synergism was performed, yielding the most conservative estimates. Follow-up studies will focus on specific products, conducting targeted investigations and in-depth toxicological interaction studies on core contaminant combinations found in high-risk varieties.

### 4.4 Limitations of cumulative risk assessment

Existing evidence suggests that combined exposure to certain heavy metals (such as cadmium) and aflatoxin B_1_ may result in synergistic hepatotoxicity and carcinogenic effects (Zhao et al., 2019). The limitation of this study lies in the use of the dose addition method to calculate the cumulative hazard index (HI), without considering the potential synergistic toxicity of co-contaminants such as heavy metals, pesticides, and mycotoxins. International authoritative institutions such as the European Food Safety Authority (EFSA, 2019) and the United States Environmental Protection Agency (USEPA, 2003) have clearly stated that in the absence of mechanism-specific interaction data and validated quantitative models, the dose addition method should be used as the default method for cumulative risk assessment. Therefore, the HI estimates in this study are conservative, aiming to prioritize the identification of high-risk variety-contaminant combinations rather than precisely quantifying absolute health risks. Future research should focus on developing interaction models based on toxicity mechanisms and cross-category toxicity equivalence factors to achieve more refined joint exposure risk assessment.

### 4.5 Limitations of risk assessment for vulnerable populations

The exposure assessment in this study was based on the average weight and daily intake of Chinese adults, and no differentiated quantitative analysis was conducted for children, the elderly, pregnant women, etc., who are vulnerable populations. The main reason is that there are currently no official statistics and usage guidelines for these special populations, making it impossible to establish standardized exposure assessment parameters. The metabolic capacity, absorption efficiency, and physiological tolerance of susceptible populations to pollutants differ from those of adults, and their actual exposure risks cannot be simply inferred by weight conversion. Take children as an example, due to the incomplete development of liver and kidney functions, their metabolic clearance ability for pollutants is weaker, and the health risks under the same exposure level may be higher. Therefore, the risk assessment results of this study mainly reflect the risk level of the adult population, and for vulnerable populations, the actual risks may differ. In the future, specific drug dosage surveys for vulnerable populations should be conducted, and combined with toxicokinetic studies, more accurate assessment of their health risks should be achieved.

## 5 Conclusion

Residues of 207 pollutants were surveyed in 42 types of TCMFGs and their potential health risks assessed. Overall risk is low, but certain varieties prone to heavy-metal accumulation or fungal contamination require attention. Aerial-part varieties accumulate heavy metals and warrant focused monitoring; underground-part varieties rich in polysaccharides are vulnerable to mycotoxins. Several banned pesticides were still detected, highlighting the need for stricter supervision. Widespread use of plant-growth regulators may affect active constituents and requires regulation. The HI-P-CV three-dimensional model established here integrates exposure, prevalence and batch-to-batch variability, converting a static risk index into a dynamic monitoring tool and providing a more scientific and accurate health-risk assessment for TCMFGs.

## Data Availability

The original contributions presented in the study are included in the article/Supplementary Material, further inquiries can be directed to the corresponding authors.
